# Supplementary figures and images for: What works, how and in which contexts when using digital health to support parents/carers to implement intensive speech and language therapy at home for children with speech sound disorder? A realist review
Source: PLoS One. 2025 May 7;20(5):e0321647. doi: 10.1371/journal.pone.0321647 (PMC12057862; doi:10.1371/journal.pone.0321647)

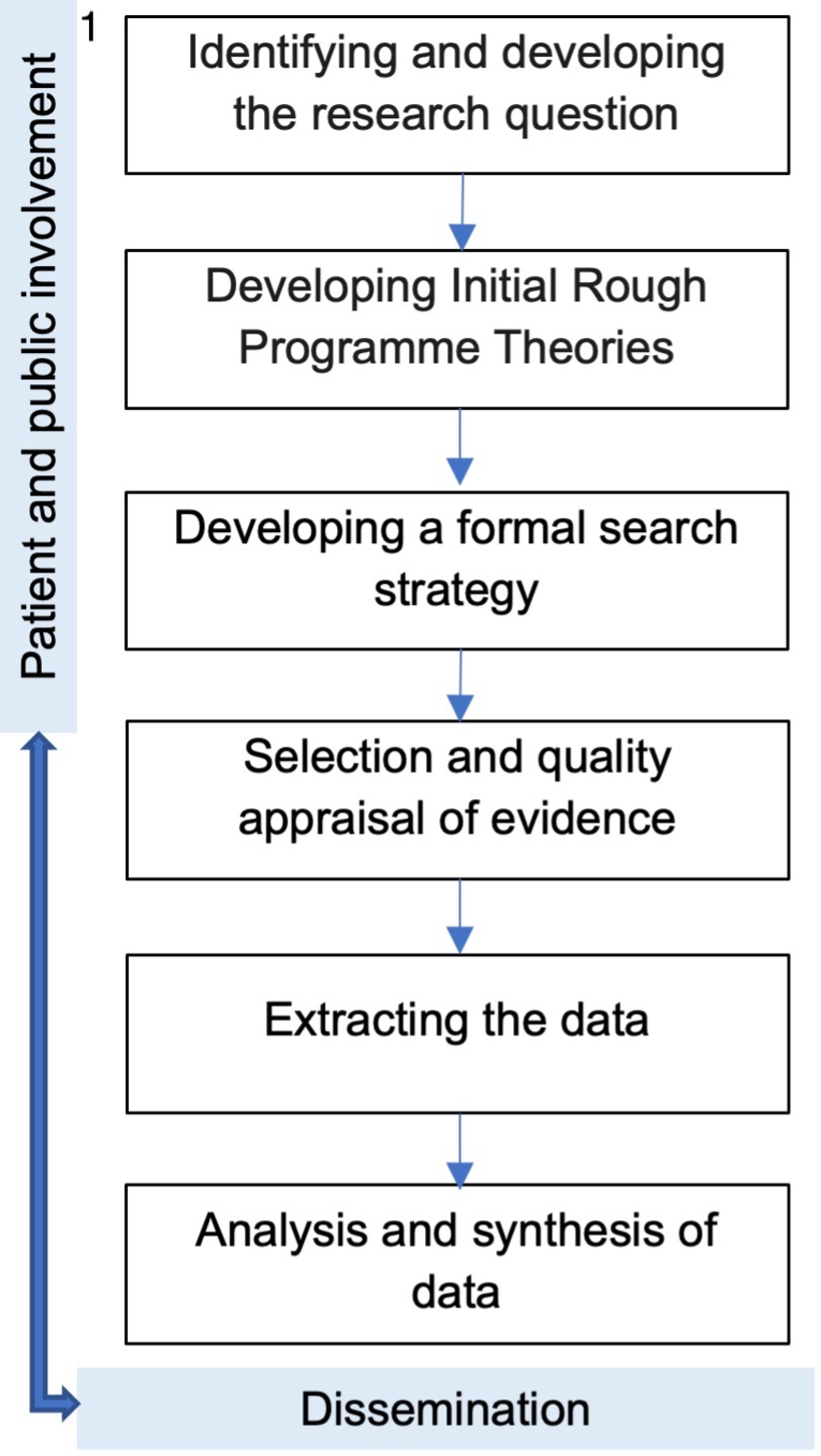

Supplement: S1 Fig — (TIF) [file pone.0321647.s001.tif]

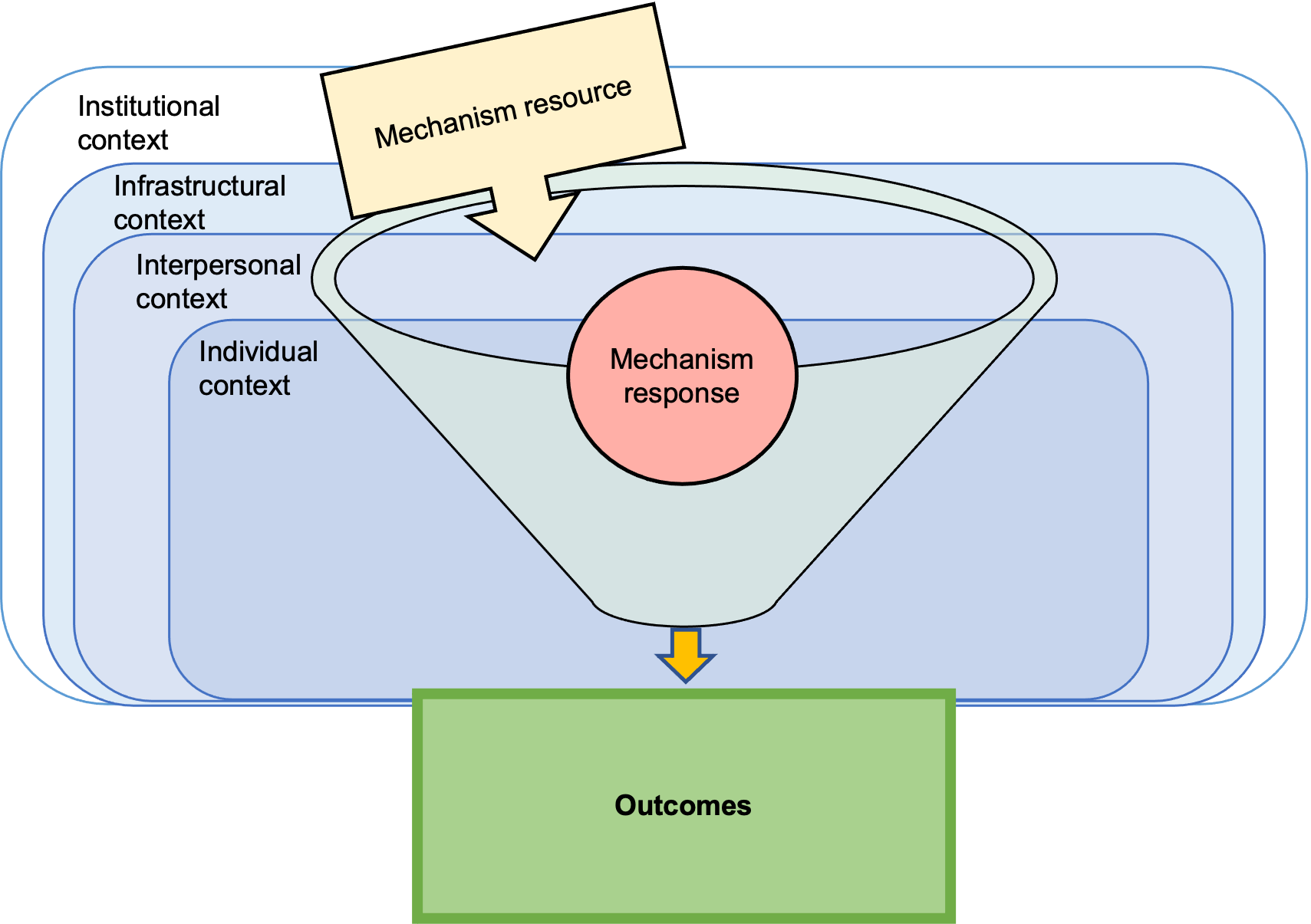

Supplement: S2 Fig — (TIF) [file pone.0321647.s002.tif]
