# Supplementary material for: What works, how and in which contexts when using digital health to support parents/carers to implement intensive speech and language therapy at home for children with speech sound disorder? A realist review
Source: PLoS One. 2025 May 7;20(5):e0321647. doi: 10.1371/journal.pone.0321647 (PMC12057862; doi:10.1371/journal.pone.0321647)
Supplement: S3 Table — (PDF) [file pone.0321647.s003.docx]

**Supplementary File 1**

*Table showing search terms using the PICO framework*

|  | **Population** | **Intervention** | **Comparison** | **Outcome** |
| --- | --- | --- | --- | --- |
| **Overview** | children with (phonological) speech sound disorder  parents/ carers | Parent- implemented interventions for speech, online speech and language therapy interventions for speech, online parent-implemented interventions, intensive parent-implemented intervention. | None / possibly to other service delivery models | Increased intervention intensity, optimise intervention efficiency, support, and empowerment for parents. |
| **Search one**  Parent-implemented speech intervention | child* OR paed*  +  speech  OR  articulat*  OR  phonolog* OR phonetic W/3  disorder*  OR  impair*  OR  difficult*  OR  problem*  OR  delay* | parent*  OR  home*  OR  carer*  W/3  implement*  OR  deliver*  OR  based  OR  practise  OR  practice  OR  homework  OR  led  OR  mediated  +  intervention OR approach* OR treatment* OR therap* OR program* | None |  |
| **Search two**  Online intervention for speech | child* OR paed*  +  speech  OR  articulat*  OR  phonolog*  OR  phonetic  W/3  disorder*  OR  impair*  OR  difficult*  OR  problem*  OR  delay* | online  OR  web*  OR  digital  OR  remote  OR  tele*  OR  computer*  OR  virtual  W/3  intervention  OR  approach  OR  treatment*  OR  therap*  OR  program*  OR  work* OR health  +  speech W/3 therap* OR patholog* | None |  |
| **Search three**  Online parent-implemented speech intervention | child*  OR  paed*  +  speech  OR  articulat*  OR  phonolog*  OR  phonetic  W/3  disorder*  OR  impair*  OR  difficult*  OR  problem*  OR  delay* | online OR  web*  OR  digital  OR  remote  OR  tele*  OR  computer*  OR  virtual  W/3  intervention  OR  approach  OR  treatment*  OR  therap*  OR  program*  OR  work*  +  parent*  OR  home*  OR  carer*  W/3  implement*  OR  deliver*  OR  based  OR  practise  OR  practice  OR  homework  OR  led  OR  mediated | None |  |
| **Search four**  Intensity and parent-implemented speech intervention | child*  OR  paed*  +  speech  OR  articulat*  OR  phonolog*  OR  phonetic* W/3  disorder*  OR  impair*  OR  difficult*  OR  problem*  OR  delay* | intensit*  OR  intensive  OR  frequen*  OR  dos*  OR  quantity  OR  amount  OR  level  W/3  intervention  OR  approach  OR  treatment*  OR  therap*  OR  program*  OR  work*  +  parent*  OR  home*  OR  carer*  W/3  implement*  OR  deliver*  OR  based  OR  practise  OR  practice  OR  homework  OR  led  OR  mediated | None |  |
| **Search five**  Online and intensive speech intervention | child* OR paed*  +  speech OR articulat* OR phonolog* OR phonetic W/3 disorder* OR impair* OR difficult* OR problem* OR delay* | online OR web* OR digital OR remote OR tele* OR computer* OR virtual W/3 intervention OR approach OR treatment* OR therap* OR program* OR work* OR health  +  AND Intensit* OR intensive OR frequen* OR dos* OR quantity OR amount OR level W/3 intervention OR approach OR treatment* OR therap* OR program* OR work* | None |  |
| **Limiters** | | | | |
| For searches completed 19.05.24:  Published between 2012-2022  Available in English  For searches completed 25.04.24:  Published between May 2022-April 2024  Available in English | | | | |
