# Supplementary material for: What works, how and in which contexts when using digital health to support parents/carers to implement intensive speech and language therapy at home for children with speech sound disorder? A realist review
Source: PLoS One. 2025 May 7;20(5):e0321647. doi: 10.1371/journal.pone.0321647 (PMC12057862; doi:10.1371/journal.pone.0321647)
Supplement: S4 Table — (PDF) [file pone.0321647.s004.docx]

| **Authors** | **Year** | **Title** | **Intervention focus relevant to research question** | **Country and language of focus** | **Study design** | **Sample size and population** | **Socioeconomic status (SES) and/or diversity** | **CMO contribution** |
| --- | --- | --- | --- | --- | --- | --- | --- | --- |
| Ahmed et al. | 2018 | Speech-driven mobile games for speech therapy: User experiences and feasibility | Digital intervention  Parent-implemented intervention  Intervention intensity | Australia  English-speaking | Pilot evaluation of use of digital mobile game – experience plus follow-up survey | 7 SLTs  10 children with CAS  6 typically developing children  Children aged 6-11 years old | Not provided | 1.3, 1.5 |
| ﻿Alighieri et al. | 2022 | The retrospective acceptability of high intensity versus low intensity speech intervention in children with a cleft palate: A qualitative study from the parents’ point of view using the Theoretical Framework of Acceptability | Intervention intensity | Belgium  Dutch-speaking | Qualitative semi-structured interviews with parents | 12 parents of 12 children with cleft palate +/- lip who had received high (n=6) or low (n=6) intensity intervention  Children aged 6-10 years old | Based on Hollingshead [1]: minimal SES score of 8 up to maximal SES score of 66  Mdn 33.42 (low intensity group)  Mdn 34.25 (high intensity group) | 3.1, 5.1, 5.2 |
| ﻿Alighieri et al. | 2021 | Is high-intensity speech intervention better? a comparison of high-intensity intervention versus low-intensity intervention in children with a cleft palate | Intervention intensity | Belgium  Dutch-speaking | Longitudinal, prospective, randomised controlled trial with a multiple baseline | PCC was compared for 12 children with cleft palate +/- lip receiving either high or low intensity intervention  Aged 6-10 years old | Based on Hollingshead [1]: minimal SES score of 8 up to maximal SES score of 66  Mdn 33.42 (low intensity group)  Mdn 34.25 (high intensity group) | 3.2, 4.5, 5.1, 5.2 |
| Allen | 2013 | Intervention efficacy and intensity for children with speech sound disorder | Intervention intensity | United States of America  American-English | Pseudo-randomised controlled trial comparing a high frequency experimental arm to a low intensity active control using multiple oppositions and a low intensity placebo story-book control group | 54 children with SSD  Aged 3-5 years  Distribution across ethnicity noted | Not provided | 5.1, 5.3, 5.4 |
| Bellon-Harn et al. | 2020 | Use of videos and digital media in parent-  implemented interventions for parents of children with primary speech sound and/or language disorders: a scoping review | Digital intervention  Parent-implemented intervention | United States of America  Papers published in English | Scoping review | Ten papers  Parents of children with primary SSD and/or language disorder  Aged 0-12 years | 5 studies did not provide SES information  5 studies provided some limited information suggesting mixed SES representation across studies | 3.1, 3.2, 4.1 |
| Crowe et al. | 2017 | Educators' perspectives on facilitating computer-assisted speech intervention in early childhood settings | Digital intervention | Australia  Australian-English | Qualitative telephone interviews following up on the experience of Early Childhood Educators participating in the Sound Start Study | 23 Early Childhood Educators (all female)  Aged 25-64 | Education levels reported from certificate qualifications to post-graduate degrees | 1.1, 1.2, 1.3, 1.4, 3.3 |
| Cummings, Giesbrecht, & Hallgrimson | 2021 | Intervention dose frequency: Phonological generalization is similar regardless of schedule | Intervention intensity | United States of America  American-English | Multiple baseline, single-participants experimental design | 8 children with SSD  Aged 4 years to 5 years and 6 months | Not provided | 5.1, 5.2, 5.3, 5.4 |
| Davies et al. | 2017 | Co-working: Parents’ conception of roles in supporting their children’s speech and language development | Parent-implemented intervention | United Kingdom  English-English | Qualitative study using semi-structured interviews | 14 parents of children with speech and language needs  Preschoolers | Low and medium SES groups | 2.2, 3.2, 4.4, 4.5, 5.2 |
| Davies et al. | 2019 | SLTs' conceptions about their own and parents' roles during intervention with preschool children | Parent-implemented intervention | United Kingdom  English-English | Qualitative study using semi-structured interviews | 12 SLTs with 6 months to 18 years experience | SLT experienced working with a diverse range of SES | 2.1, 3.2, 4.3, 4.5 |
| Doube et al. | 2018 | Comparing feedback types in multimedia learning of speech by young children with common speech sound disorders: Research protocol for a pretest posttest independent measures control trial | Digital intervention  Parent-implemented intervention | Australia  Australian-English | Protocol for a pre-test post-test independent measures control trial | Population to be targeted:  Children with phonological delay and disorder  Aged 4-6 years | Not considered in protocol | 1.4, 3.2, 3.3 |
| Fairweather, Lincoln, & Ramsden | 2016 | Speech-language pathology teletherapy in rural and remote educational settings: Decreasing service inequities | Digital intervention | Australia  Australian-English | Group treatment study using mixed method evaluation through collection of post-intervention quantitative and qualitative data. | 19 children  Aged 3-12 years  5 parents | Diverse ethnicity represented in the child population but not the parent population  47.4% of children were Aboriginal or Torres Strait Islanders | 1.1, 4.2, 5.2 |
| Furlong et al. | 2018 | Processes and challenges in clinical decision-making for children  with speech-sound disorders | Digital intervention  Parent-implemented intervention  Intervention intensity | Australia  Australian-English | In-depth semi-structured individual interviews | 11 SLTs who worked with children with SSD | Some SLTs worked with children of low SES and diverse backgrounds | 1.1, 1.2 3.1, 3.2, 4.1, 4.2, 4.4, 4.5, 5.1, 5.2 |
| Gacnik et al. | 2018 | User-centred app design for speech sound disorders interventions with tablet computers | Digital intervention | Slovenia  Language not stated but potentially Slovenian | Mixed research methodology to develop and evaluate a prototype for an app  72 SLTs completed a general survey on their use of digital tools in clinical practice to inform the development of a prototype app  5 SLTs contributed to the development of the app through interviews and trialled the prototype with 82 children | 72 SLTs completed survey  5 female SLTs (with 2-36 years experience) co-produced and tested the prototype with 82 children with SSD  Children aged 3 years and 6 months to 11 years and 11 months | Not provided | 1.1, 1.4, 5.2 |
| Hair et al. | 2021 | A longitudinal evaluation of tablet-based child speech therapy with apraxia world | Digital intervention  Parent-implemented intervention  Intervention intensity | Australia  Australian-English | Longitudinal, quantitative descriptive study  Evaluated improvement in pronunciation, engagement of child, accuracy of caregiver and automated pronunciation judgements after two counter-balanced 4-week treatment blocks divided by a 2-week break. | 11 children with SSD (and their parents)  Aged 5-12 years | Not provided | 1.1, 1.3, 1.4, 1.5 |
| Hair et al. | 2018 | Apraxia world: A speech therapy game for children with speech sound disorders | Digital intervention | Australian  Australian-English | Within subject study design evaluating two versions of Apraxia World to see how two approaches to delivering speech exercises impacted child engagement and outcome | 21 children  14 children with SSD  7 reported to be typically developing  Aged 4-12 years | Not provided | 1.1, 1.4, 1.5 |
| Hegarty, Titterington, & Taggart | 2021 | A qualitative exploration of speech-language pathologists’ intervention and intensity provision for children with phonological impairment | Intervention intensity | United Kingdom  English-English and Irish-English | Focus groups with SLTs  1:1 interviews with SLT managers | 15 SLTs (0-30 years of experience)  6 SLT managers (11-35 years of managerial experience) | Not provided | 4.1, 5.1 |
| Hodge & Gaines | 2017 | Pilot implementation of an alternate service delivery model for young children with severe speech and expressive language delay | Parent-implemented intervention  Intervention intensity | Canada  Canadian-English | Case series pre-post design (with no control).  Report on a pilot implementation of a novel service delivery model. The model provided 1:1 training of parents including goal setting, followed by treatment provided twice weekly over 8 weeks | 10 child-parent dyads  Children with expressive language and speech delay  Aged 2 years and 10 months to 3 years and 7 months old | Not provided | 1.2, 1.5, 2.3, 3.2, 3.3, 4.1, 5.4 |
| Jesus et al. | 2019 | Comparing traditional and tablet-based intervention for children with speech sound disorders: A randomized controlled trial | Digital intervention | Portugal  Portuguese | Randomised controlled trial | 22 Children with phonologically based SSD  Aged 4 years and 7 months (mean) | Not provided | 1.1, 1.4, 1.5 |
| Jesus, Santos, & Martinez | 2019 | The table to tablet (T2T) speech and language therapy software development roadmap | Digital intervention | Portugal  Portuguese | Roadmap development of software documenting the table to tablet process  A design-based research method | Development phase – 2 SLTs, 2 software developers, a designer and a speech scientist.  Pre-test phase: 7 children with SSD  Mean age 4 years and 8 months  Software development:  22 children  Aged between 3 years and 5 months to 6 years and 5 months  Activities development: same team as development phase | Not provided | 1.1, 1.4 |
| Klatte et al. | 2020 | Collaboration between parents and SLTs produces optimal outcomes for children attending speech and language therapy: Gathering the evidence | Parent-implemented intervention | Netherlands, UK and Ireland  English | Realist evaluation approach  Involving discussion and agreement within a research group of 7 academics specialising in collaborative practice in SLT  Informal review of several relevant papers  Development of theory and recommendations for practice | 7 academics specialising in collaborative practice | Not provided | 3.2, 4.3, 4.5 |
| Lim, McCabe, & Purcell | 2020 | Look at Mummy: Challenges in training parents to deliver a home treatment program for childhood apraxia of speech in a rural Canadian community | Parent-implemented intervention | Canada and Australia  Canadian-English | Experimental single-case across behaviours design  Parent interviews | 4 parent-child dyads  Children met criteria for CAS  Aged 3-8 years | Mixed SES | 1.1, 1.2, 2.1, 2.2, 4.4, 5.2 |
| McCormack et al. | 2017 | Implementation fidelity of a computer-assisted intervention for children with speech sound disorders | Digital intervention  Intervention intensity | Australia  Australian-English | Comparison of three datasets from an earlier RCT to examine fidelity of Educators’ implementation of the Phoneme Factory in the Sound Start study | Data reported for 63 children (4;01-5;05) with SSD across 19 early childhood centres | Range of SES | 3.1, 3.2, 5.1 |
| McFaul et al. | 2022 | Applying evidence to practice by increasing Intervention intensity for children with severe speech sound disorder: A quality improvement project | Intervention intensity | Northern Irish  Irish-English | Quality improvement project evaluating implementation of evidence-based intensity of intervention | 10 children with severe SSD  Aged 4-5 years old | Not provided | 5.1, 5.2, 5.3 |
| McKechnie et al. | 2020 | The influence of type of feedback during tablet-based delivery of intensive treatment for childhood apraxia of speech | Digital intervention  Intervention intensity | Australia | Parallel group design comparing two types of feedback | 14 children with CAS  Aged 4-10 years old | Not provided | 3.2, 3.3, 5.1 |
| McLeod et al. | 2017 | Cluster-randomized controlled trial evaluating the effectiveness of computer-assisted intervention delivered by educators for children with speech sound disorders | Digital intervention  Parent-implemented intervention | Australia  Australian-English | Cluster-randomized controlled trial | 123 children with SSD  39 early childhood centres  Aged 4.0-5.04 years | Range of SES | 1.2, 5.1 |
| McLeod et al. | 2023 | ﻿Equitable access to speech practice for rural Australian children using the SayBananas! mobile game | Digital intervention  Intervention intensity | Australia  Australian-English | Mixed methods | 45 children with SSD  Aged 4;04-10;05 years | Not provided  From rural areas of Australia | 1.1, 1.2, 1.5, 3.2, 5.1, 5.3, 5.4 |
| McLeod et al. | 2020 | Waiting for speech-language pathology services: A randomised controlled trial comparing therapy, advice and device | Digital intervention | Australia  Australian-English | Four-stage randomised controlled trial | 110 children with significant speech/language delay/disorder  Aged 3-6 years old | 14.9% of children identified as Aboriginal or as Torres Strait Islanders | 3.2, 5.1 |
| Namasivayam et al. | 2019 | Investigating intervention dose frequency for children with speech sound disorders and motor speech involvement | Intervention intensity | Canada  Canadian-English | Pre-post design with stratified randomisation | 48 children with motor SSD  Aged 3;06-3;09 | Not provided | 1.5, 4.1, 5.1, 5.3 |
| Namasivayam et al. | 2023 | ﻿Predictors of Functional Communication Outcomes in Children with Idiopathic Motor Speech Disorders | Parent-implemented intervention  Intervention intensity | Canada  Canadian-English | Pre-post design with stratified randomisation | 85 children with SSD – 37 with CAS and 48 with speech motor delay  Aged 2;05-5;02 | Not provided | 5.1, 5.4 |
| Parnandi et al. | 2015 | Development of a remote therapy tool for childhood apraxia of speech | Digital intervention | Unclear  (across US, Qatar and Sydney) | Software development and its piloting | 8 children with CAS  Aged 4-10 years | Not provided | 1.1, 1.2, 1.5 |
| Rvachew & Brosseau-Lapré | 2015 | A randomized trial of 12-week interventions for the treatment of developmental phonological disorder in francophone children | Intervention intensity  Parent-implemented intervention | Canada  French-Canadian | Randomised controlled trial comparing input vs output oriented therapy | 72 children with SSD (phonological disorder)  4 years old | Limited information on mothers’ education status | 4.1, 5.1, 5.3, 5.4 |
| Santos et al. | 2022 | Home-based activities for children with speech sound disorders: requirements for a tangible user interface for internet of things artefacts | Digital intervention  Intervention intensity | Portugal  Portuguese | Systematic literature review, focus group and nationwide questionnaire to develop requirements for an app to support children with SSD | One focus group of 5 specialists (SLTs/Human-computer interaction professionals).  33 SLTs | Not provided | 1.1, 1.4, 4.1, 4.2, 4.3, 4.4, 4.5, 5.2 |
| Sell et al. | 2023 | Parent experiences of undertaking therapy for cleft palate speech disorders following in-depth training | Digital intervention  Parent-implemented intervention | UK and Ireland  English-English  Irish-English | Qualitative focus groups and telephone interviews | 18 parents (of children with cleft related SSD) | Range of nonmanual to professional occupations.  Educational achievement ranged from A level/leaving cert to doctoral level (7 parents has MSc level and 3 PhD level education) | 1.1, 1.3, 1.5, 2.1, 2.2, 3.1, 3.2, 4.1, 4.4, 5.3 |
| Silva et al. | 2023 | ﻿Efficacy in the use of gamification strategy in phonological therapy | Digital intervention | Brasil  Brazilian-Portuguese | Prospective and longitudinal study comparing traditional phonological therapy to therapy using gamification | 10 children with phonological disorder  Aged 4-8 years | Not provided | 1.1, 1.5, 4.3 |
| Sugden et al. | 2018 | An Australian survey of parent involvement in intervention for childhood speech sound disorders | Parent-implemented intervention | Australia  Australian-English | Survey | 288 SLTs working with children with SSD | Less than 10% of caseloads were from culturally and linguistically diverse backgrounds | 1.1, 4.2, 4.5, 5.1, 5.2 |
| Sugden et al. | 2020 | Evaluation of parent- and speech-language pathologist-delivered multiple oppositions intervention for children with phonological impairment: A multiple-baseline design study | Parent-implemented intervention | Australia  Australian-English | Multiple baseline across participants design | 5 children with moderate-severe phonological impairment  Aged 3;3-5;11 | Range of SES represented | 1.2, 4.4, 4.5, 5.1 |
| Sugden et al. | 2019 | Parents' experiences of completing home practice for speech sound disorders | Parent-implemented intervention | Australia  Australian-English | Qualitative semi-structured interviews | 6 parents of children with SSD  Children aged 3-6 years | Range of SES represented | 2.1, 2.2, 2.3, 3.2, 4.1, 4.2, 4.4, 4.5, 5.1, 5.2 |
| Sweeney et al. | 2020 | Randomized controlled trial comparing Parent Led Therapist Supervised Articulation Therapy (PLAT) with routine intervention for children with speech disorders associated with cleft palate | Digital intervention  Parent-implemented intervention | UK and Ireland  English-English  Irish-English | Randomised controlled trial comparing PLAT (parent-led therapist supervised articulation therapy) with routine care for children with Cleft-related SSD | 53 children with Cleft-related SSD  Aged 2.9-7.5 years | Parents education level represented | 4.1, 4.2, 4.5 |
| Tambyraja | 2020 | Facilitating parental involvement in speech therapy for children with speech sound disorders: A survey of speech-language pathologists’ practices, perspectives, and strategies | Parent-implemented intervention | United States  American-English | Survey | 156 SLTs working with children with SSD | Not provided | 3.1, 3.2, 4.2, 4.3 |
| Thomas, McCabe & Ballard | 2018 | Combined clinician-parent delivery of rapid syllable transition (ReST) treatment for childhood apraxia of speech | Parent-implemented intervention | Australian  Australian-English | Multiple baseline across participants design | 5 children with CAS  Aged 5;1-11;7 | Not provided | 2.1, 3.2, 3.3 |
| Thomas et al. | 2018 | Parent experiences of variations in service delivery of Rapid Syllable Transition (ReST) treatment for childhood apraxia of speech | Parent-implemented intervention | Australian  Australian-English | Qualitative semi-structured interviews | 10 parents of children with CAS | Range of urban/rural areas | 2.2, 3.2, 3.3, 4.4, 5.1, 5.2 |
| Tosh, Arnott & Scarinci | 2017 | Parent-implemented home therapy programmes for speech and language: a systematic review | Parent-implemented intervention | Australia  Australian-English | Systematic review | Parent-implemented intervention for children 2+years of age with speech and/or language difficulties |  | 2.1, 3.2, 4.5, 5.1 |
| Watts Pappas, McAllister, & McLeod | 2016 | Parental beliefs and experiences regarding involvement in intervention for their child with speech sound disorder | Parent-implemented intervention | Australia  Australian-English | Multiple sequential interviews during intervention | 7 parents of 6 children with SSD (21 interviews in total)  Children aged 3;0-5;1 | Education level ranged from high school to university level | 2.2, 2.3, 3.1, 3.2, 4.1, 4.5 |

References

1. Hollingshead AB. Four factor index of social status [Internet]. 1975
