# Supplementary material for: What works, how and in which contexts when using digital health to support parents/carers to implement intensive speech and language therapy at home for children with speech sound disorder? A realist review
Source: PLoS One. 2025 May 7;20(5):e0321647. doi: 10.1371/journal.pone.0321647 (PMC12057862; doi:10.1371/journal.pone.0321647)
Supplement: Attachment — Submitted filename: Response to reviewers.docx [file pone.0321647.s007.docx]

Dear Professor Sandeep Reddy,

Thank you for taking the time to review our manuscript and for the constructive and helpful comments from the reviewers. Please see our responses to each comment in the table below, with details of the relevant revisions made to the manuscript.

**Response to reviewers’ comments**

| **Journal requirements** | **Response** |
| --- | --- |
| 1. Please ensure that your manuscript meets PLOS ONE's style requirements, including those for file naming. | The manuscript has been checked to ensure it meets PLOS ONE’s style requirements and minor amendments made (marked as tracked changes).  File names have been amended for figures, tables, and supporting information. |
| 2. Thank you for stating in your Funding Statement: "This review has been completed as part of a PhD studentship at Ulster University (NL) which is supported by the Department for the Economy (DfE). The funders had no role in study design, data collection and analysis, decision to publish, or preparation of the manuscript."   Please provide an amended statement that declares *all* the funding or sources of support (whether external or internal to your organization) received during this study, as detailed online in our guide for authors. Please also include the statement “There was no additional external funding received for this study.” in your updated Funding Statement.  Please include your amended Funding Statement within your cover letter. We will change the online submission form on your behalf. | Please see amended statement below:  *All the funding for this review has come from a PhD studentship at Ulster University (NL) supported by the Department for the Economy (DfE). The funders had no role in study design, data collection and analysis, decision to publish, or preparation of the manuscript. There was no additional external funding received for this study.* |
| 3. In the online submission form, you indicated that [The data underlying the results presented in the study are available from Professor Laurence Taggart (l.taggart@ulster.ac.uk).].  All PLOS journals now require all data underlying the findings described in their manuscript to be freely available to other researchers, either 1. In a public repository, 2. Within the manuscript itself, or 3. Uploaded as supplementary information. This policy applies to all data except where public deposition would breach compliance with the protocol approved by your research ethics board. If your data cannot be made publicly available for ethical or legal reasons (e.g., public availability would compromise patient privacy), please explain your reasons on resubmission and your exemption request will be escalated for approval. | The full data set will be uploaded to Ulster University’s data repository which includes the full set of Context-Mechanism-Outcome configurations (CMOs) with supporting literature, and example quotations from included literature. |
| 4. When completing the data availability statement of the submission form, you indicated that you will make your data available on acceptance. We strongly recommend all authors decide on a data sharing plan before acceptance, as the process can be lengthy and hold up publication timelines. Please note that, though access restrictions are acceptable now, your entire data will need to be made freely accessible if your manuscript is accepted for publication. This policy applies to all data except where public deposition would breach compliance with the protocol approved by your research ethics board. If you are unable to adhere to our open data policy, please kindly revise your statement to explain your reasoning and we will seek the editor's input on an exemption. Please be assured that, once you have provided your new statement, the assessment of your exemption will not hold up the peer review process. | See point 3 above. Data will be available through Ulster University’s data repository at the time of publication. |
| 5. Please review your reference list to ensure that it is complete and correct. If you have cited papers that have been retracted, please include the rationale for doing so in the manuscript text, or remove these references and replace them with relevant current references. Any changes to the reference list should be mentioned in the rebuttal letter that accompanies your revised manuscript. If you need to cite a retracted article, indicate the article’s retracted status in the References list and also include a citation and full reference for the retraction notice. | The reference list has been checked for accuracy and formatting, and required amendments made. New references have been added relevant to suggested revisions. Any references noted be missing in error have been added and marked as tracked changes in the document. No retracted papers have been cited to our knowledge. |

| **Reviewer #1 comments** | **Response** |
| --- | --- |
| The method is robust and well-structured, uses the realist review approach to explore the complexities of parent-implemented speech and language interventions. Moreover, the use of a realist review is particularly commendable. It goes beyond traditional review approaches by focusing on understanding the underlying mechanisms of how, why, and in what contexts these interventions are most effective. | Thank you for taking the time to review the paper and for your positive review and comments, it is much appreciated. |

| **Reviewer #2 comments** | **Response** |
| --- | --- |
| General comments:  Thank you for the opportunity to read this article. I found this paper of great relevance to clinical practice and the field of research. I congratulate the authors on a well-organised, clearly written paper with excellent objectives that hold importance and application to modern day work with families. Tables 4 to tables 8 alone are a fantastic resource for the field internationally. It is clear a lot of work has gone into this publication.  The only question I feel you have not answered is for whom (see abstract, line 41) – for which parent/carers and for which children with SSD might these parent-implemented digital innovations work for? Do all your papers sample white, middle-high class, well educated, English speaking mothers? That’s an important lens to raise in your conclusion and in your limitations as it has implications for how SSD interventions will work with underserved / under researched groups, i.e., fathers, global majority families, low SES families living in and out of western, education, industrialised, rich and democratic (WEIRD) countries.  Further, whilst I appreciate you have a published protocol, I feel a bit more detail is required in this manuscript. Not everyone, especially clinicians and non-SLT readers, will have capacity to read both and you are doing your brilliant work a disservice by not including single sentences to describe important decisions such as your exclusion/inclusion criteria | Thank you for your positive feedback on our manuscript and for your helpful, constructive comments and suggestions for revisions. We agree that these revisions will help strengthen the work and add clarity for readers. The specific revisions made to the manuscript are outlined in the points below.    Further information about study characteristics has been added to the table of included literature, including samples included in studies (see S4 Table). Reference to individual family contexts that impact on outcomes identified through the review has been added to p.43, line 953-955. The possible implications of the samples included and the importance of this lens on theory development and future research has been acknowledged in the study limitations (p.44, lines 967-977).  Further detail has been added to the methods of the manuscript as suggested. Details of these changes are outlined in the points below. |
| **Introduction** | |
| 1. Strong introduction that clearly builds your argument for the work. As PLOS ONE is a generalist journal, I suggest a definition of SSD, levels of incidence, and perhaps a descriptor of how a child with a ‘moderate’ or ‘severe’ SSD would present. | A definition and prevalence rates have been added to p.4, line 47-54. |
| 2. Table 1, page 5. Please share some participant demographics (child age, SES, languages spoken) on these studies – see point above ‘for whom’. | Please see the amended Table 1, p.5 which now includes information on these areas either directly in the table or in the note below it. |
| 3. Line 166, p 8 of the manuscript: ‘Bellon-Harn et al. [29] studied the use of videos/digital media in parent167 implemented interventions for children with primary SSD or language disorder’. Can you add HOW Bellon-Harn and colleagues did this, i.e. was it an RCT? etc. | Information has been added to p.9 lines 179-180 (this was a scoping review) |
| **Method** | |
| 4. Add one sentence on why your team selected a realist review as a method to this manuscript | A sentence has been added to p.11, line 223-225 to explain why a realist review was selected for the method of this manuscript. |
| 5. P 10. Again, for time-poor readers unable to read your protocol paper, I would add your inclusion and exclusion criteria for papers to your methods. I only noticed your criterion in your supplementary files, i.e., ages 2-7, no comorbidities. | Information about the inclusion and exclusion criteria has been added to p.13 in Table 3. |
| 6. p. 14, line 243 ‘explicit or implicit references to relevant MRTs were noted, then explored in depth, and tested using evidence extracted from the literature’. Please provide one example of an MRT. Perhaps better added in Table 2 – as you provide examples of other definitions here already. | Thank you for this useful point. An example of an existing MRT has been added to the MRT definition in Table 2, p.12. |
| 7. p.15, line 258. I wondered if one or two of your IMRTs could be listed, so early thinking is shared. I don’t see these in your protocol with a quick glance. | Please see Table 3, p.13, where an example of an Initial Rough Programme Theory (IRPT) has been added as suggested. |
| 8. Your s1 flowchart labels ‘PPI’ but your manuscript does not refer to PPI. Please add a PPI paragraph to describe those involved and how they shaped the review. Did they refine your RQ for example? Who were they? Also, is PPI the same as your expert steering group? Who were they? Any experts with lived experience? | Thank you for your suggestion. A paragraph to explain PPIE (through the expert steering group involvement) has been added to p.16, lines 285-295. |
| **Results** | |
| 9. I would perhaps consider adding the table of included publications to the paper (rather than a supplementary file) and to save space, remove the title of the paper and instead report on some other characteristics such as country of study, research design, sample size of each study, SES, quality rating, maybe even some cross referencing to tables 4 – 8 i.e. which papers contributed to which CMOs (i.e. paper 1 Amed et al 2018 – CMOs 1.1, 1.4, etc) | In response to this helpful suggestion, the table of included studies has been amended to report on key study characteristics most relevant to the realist review, e.g., country of study, design, sample size and characteristics, SES where included, and cross reference to contribution to CMOs.  The new level of detail included in the table means we have been unable to add the table to the main manuscript as it is now 12 pages long. Therefore, we feel it should still be included as a supplementary file (S4 Table) and this has been referenced within the main text. For this reason, we have still included the titles of the papers to support clarity in the supplementary file. When completing the table, we noted that one paper had been omitted in error and this has been corrected. |
| 10. I would also consider listing your CMOs/programme areas in a different priority – first centring the child (participation), then parent, parent/SLT collab, and ending with intensity. This would be in line with Brofenbrenner’s theory of the child first, within a context of their family and community.  Whilst reading through CMO 2.2 I kept wanting to read about the variable partnership between parent and child with SSD? What about their interaction, communication, temperament when without the SLT present and trying to work on SSD Tx? Much later, I then read CMO 4.2. It made me think intensity is irrelevant if the child will not participate, hence the re-structure suggestion. | Thank you for this helpful suggestion. The order of programme areas has been amended in the manuscript as suggested to include the programme theory areas in the following order:  1. Child-participation  2. The child-parent-SLT dynamic  3. Parent-training  4. Partnership and collaboration  5. Intervention intensity |
| 11. Fig 2 – Are the cogs different sizes because they hold different weight/significance? Can you number the cogs so they match up with your programme areas? | Fig 2 has been amended so that the cogs are now equally sized. A figure legend has been added (see Figure Legends p.53) to describe the link between the cogs and the five programme areas. |
| 12. For ‘CMO 2.3: Individual and service level contexts impact on SLT confidence….’ You say ‘Literature suggested that SLTs’ working contexts…’ – can you qualify – in which countries? I sense UK studies, but is this a global (WEIRD countries) phenomenon? | Thank you for raising this point. Clarification of the countries of cited studies has been added on p. 33, line 689 (now CMO 4.3). |
| **Discussion** | |
| 13. It would be great to see more of the wider literature on digital health tools / behaviour change.   Could it be worth looking at Behaviour Change Techniques and Mechanisms of Action and weaving some of these through your discussion – see <https://pmc.ncbi.nlm.nih.gov/articles/PMC6636886/>  For example - P. 37, Line 795-796 ‘…how behaviour change theories (which ones, add references) and adult-learning theory (which ones, add references) underpin this complex intervention’. | Thank you for this suggestion. An overview of current literature on digital health in speech and language therapy is provided in the introduction (p.8-10) and we discuss this literature in the results and discussion (for example p.42, lines 910-923). A key learning point from this review is that the mechanisms involved in intervention delivered through digital tools or face to face are very similar, described in the discussion. We do not feel we have space to go into more detail about digital health in this section. See points below about behaviour change.  Thank you for recommending this paper by Carey et al. (2018). This was an interesting and valuable paper that we enjoyed reading. We felt there was a crossover between the behaviour change techniques and mechanisms of action outlined in the suggested paper and the theoretical thinking developed in this review.  In realist science, the mechanism is a unique combination of the resources offered by the intervention and the responses of those receiving the intervention. We have attempted to make the realist terminology around mechanisms much clearer in the methods section and throughout (see methods p.15, lines 236-237 and 247-251, and discussion p.39, lines 834-844). The example model of a CMO configuration showing resource and response mechanisms has been moved to earlier in the manuscript from p.20 to p.15 (line 252) for further clarity. This has been added as a supporting file (S2 Figure) to meet PLOS ONE’s requirements of Fig 1 being the flow diagram for literature searches and selection. It would be easier for the reader if S2 Figure could be embedded in the text as Fig 1 if possible.  Behaviour change theory has been used across all aspects of the realist review to help develop theoretical thinking, specifically linked to the BCW and TDF, which is described in the methods (p.18) and discussion (p.39-41). Further clarity and detail of the specific behaviour change theories and adult-learning theory used to help develop theoretical thinking has been added to the methods (p.18, lines 318) and discussion (p.39), lines 834-844. |
| **Limitations** | |
| 14. See first point – you mention ‘for whom’ but I think more detail needs to be added here, rather than ‘all parents’ and ‘all children with SSD’ – is there bias within the recruitment of your included studies? | Thank you for this suggestion. As indicated above in the response to general comments, further detail has been added to the limitations section to address this important point (see p.44, line 967-977). |

| **Additional minor changes made to manuscript** | |
| --- | --- |
| **Change made** | **Explanation** |
| 1. Supporting information file 3 (S3 Table - Bespoke Tools) removed. | The bespoke tools for identification, selection, appraisal, and data extraction were included in the original submission as a supporting file, however these tools are already published in the realist review protocol paper and therefore need to be removed from this manuscript. These files can be found in the published protocol (Leafe et al. 2024), which is referenced within the text.  Supporting information files have been renamed accordingly. |
| 2. Minor amendments made to the manuscript (e.g., formatting, clarity of sentences), marked as tracked changes | Minor amendments made as required to support clarity of the manuscript.  Figure numbers have been amended throughout the text and in Figure Legends due to the suggested revisions made. |

We look forward to hearing from you.

Yours sincerely,

Naomi Leafe (corresponding author)
